# Two vs three cycles of neoadjuvant immunochemotherapy for resectable non-small-cell lung cancer: a real-world population-based study

**DOI:** 10.3389/fimmu.2025.1654830

**Published:** 2025-11-17

**Authors:** Jiawei Xiu, Xin Yao, Ying Sun, Shaopeng Xu, Chao Wang, Hongtao Duan, Xiaolong Yan

**Affiliations:** Department of Thoracic Surgery, The Second Affiliated Hospital, Air Force Medical University, Xi’an, China

**Keywords:** immunotherapy, neoadjuvant immunochemotherapy (NICT), NSCLC, cycle, duration (time), neoadjuvant therapy

## Abstract

**Objective:**

Investigation of the Impact of Neoadjuvant Immunochemotherapy Cycles on Pathological Response, Perioperative Safety, and Survival Outcomes in Patients with Resectable Non-Small Cell Lung Cancer (NSCLC).

**Methods:**

This study utilized real-world data, focusing on patients with stage IIA-IIIB non-small cell lung cancer (NSCLC) who underwent neoadjuvant immunochemotherapy followed by surgical resection. Subjects were stratified into groups based on whether they received two or three cycles of neoadjuvant therapy. Propensity score matching (PSM) and inverse probability weighting (IPW) analyses were utilized to adjust for covariates, thereby balancing seven clinically relevant variables, including demographic factors, and tumor characteristics, to ensure baseline comparability. Following the application of PSM and IPW, comparisons were conducted between the two-cycle and three-cycle groups in terms of pathological response indicators [pathological complete response (pCR) and major pathological remission (MPR)], perioperative safety metrics, and survival outcomes [overall survival (OS) and disease-free survival (DFS)].

**Results:**

pCR rates were comparable between the three-cycle and two-cycle groups both before adjustment (40.2% vs 42.0%; OR = 0.93, *P* = 0.777) and after PSM (48.1% vs 42.0%; OR = 1.28, *P* = 0.430) or IPW (42.0% vs 43.7%; OR = 0.93, *P* = 0.801). Similarly, MPR rates showed no significant differences (pre-adjustment: 63.8% vs 70.4%, *P* = 0.283; PSM: 66.7% vs 70.4%, *P* = 0.612; IPW: 64.6% vs 69.5%, *P* = 0.440). Perioperative safety profiles were comparable. After median follow-ups of 25.3 (three-cycle) and 31.3 (two-cycle) months, three-year DFS (84.6% vs 88.2%; HR = 1.04, *P* = 0.921) and OS (88.6% vs 88.2%; HR = 0.94, *P* = 0.892) were not significantly different. Achieving MPR or pCR was independently associated with significantly improved DFS (MPR: HR = 0.25, *P* < 0.001; pCR: HR = 0.25, *P* = 0.005) and OS (MPR: HR = 0.30, *P* = 0.002; pCR: HR = 0.28, *P* = 0.018) compared to non-responders.

**Conclusion:**

Our analysis demonstrated comparable pathological responses (pCR/MPR) between 2-cycle and 3-cycle neoadjuvant immunochemotherapy.

## 1 Introduction

In the past decade, there has been a proliferation of novel neoadjuvant treatment modalities in non-small cell lung cancer (NSCLC) (1). The publication of the CheckMate 816 trial results in 2022 marked the beginning of a new era for neoadjuvant immunochemotherapy in resectable NSCLC (2). Shortly thereafter, the NCCN updated its guidelines based on these findings, recommending nivolumab in combination with platinum-based doublet chemotherapy as a neoadjuvant immunotherapy regimen for specific patient populations (those with tumors ≥ 4 cm or lymph node positivity, and without contraindications to immune checkpoint inhibitors) (3). The efficacy and safety of neoadjuvant treatment modalities have been validated through phase III clinical trials. However, there remains no consensus on the optimal duration for neoadjuvant therapy. Both the CheckMate-159 and LCMC3 trials have utilized two-cycle immunotherapy regimens (4, 5). Additionally, three-cycle neoadjuvant immunotherapy regimens have been extensively employed in clinical trials (1). Regarding the optimal treatment duration for neoadjuvant therapy, specifically whether to use 2 or 3 cycles, Miner Shao et al. conducted the neoSCORE clinical trial in 2023. The study reported firstly that 3 cycles of neoadjuvant therapy resulted in a numerically higher major pathological response (MPR) rate compared to 2 cycles (41.4% vs 26.9%; *P* = 0.26) (6). Given these findings, additional evidence is required to further elucidate this issue. To address this gap, the author employed propensity score matching (PSM) and inverse probability weighting (IPW) to compare pathological remission, perioperative safety, and survival outcomes between 2-cycle and 3-cycle neoadjuvant therapy regimens.

## 2 Methods

### 2.1 Data and study population

**Figure 1:**
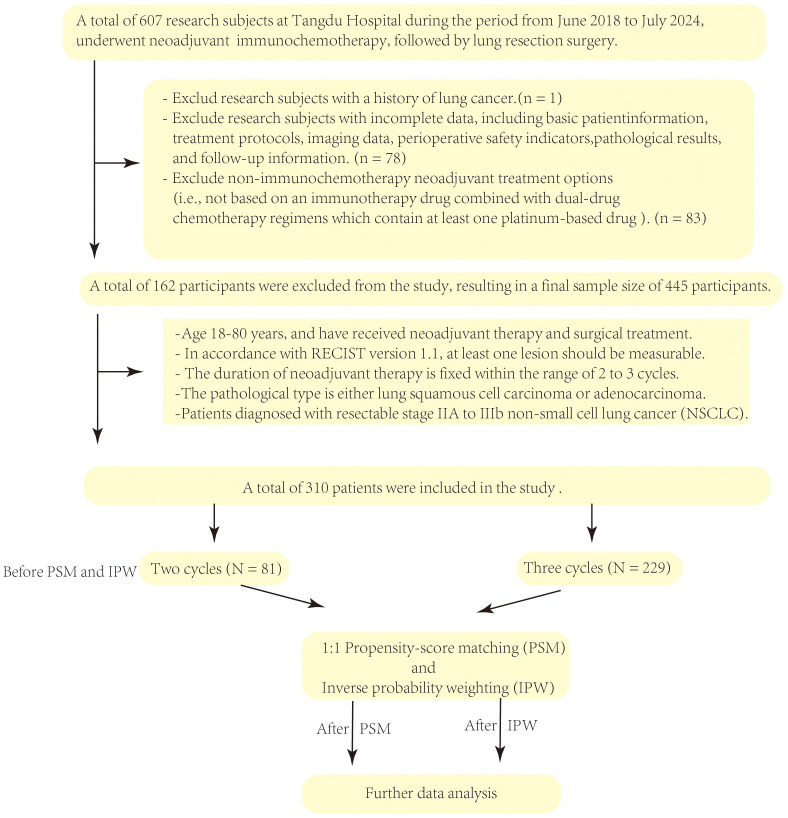
Flowchart of the research subject selection process.

The study population consisted of patients aged 18–80 years with a cytologic/histologic diagnosis of NSCLC and underwent surgery following neoadjuvant immunochemotherapy at the Department of Thoracic Surgery, Tangdu Hospital, Fourth Military Medical University, between January 2018 and November 2023. All patients underwent an extensive series of baseline imaging examinations, including chest computed tomography (CT), head CT, whole-body bone scans, abdominal ultrasound, and positron emission tomography-computed tomography (PET-CT), to rule out the presence of distant metastases. Inclusion criteria also required that patients had at least one measurable primary lesion according to Response Evaluation Criteria in Solid Tumors (RECIST, version 1.1) (7). Neoadjuvant immunochemotherapy was administered in 2–3 cycles. The pathological types included lung squamous cell carcinoma and lung adenocarcinoma, with clinical stages ranging from IIA to IIIB. Exclusion criteria were as follows: patients with a prior history of lung cancer; patients with incomplete patient, clinical, pathological, or follow-up data; patients whose neoadjuvant treatment regimen did not conform to a combination of an immune checkpoint inhibitor plus a platinum-based doublet chemotherapy. This study was approved by the local ethics committee, with the approval number being (K-HG-202506-04). This study was approved by the Institutional Review Board (Approval No. K-HG-202506-04) and granted a waiver of informed consent due to its retrospective design. The selection process for research subjects is illustrated in **Figure 1**.

### 2.2 Data acquisition and definitions

Patient baseline data, medical information during neoadjuvant therapy, and perioperative information were obtained from electronic medical records, electronic imaging records, prescription records, and other relevant documents during their treatment in our department. Preoperative clinical staging (cTNM stage) was determined according to the eighth edition of the American Joint Committee on Cancer (AJCC) lung cancer cTNM staging system and confirmed by an independent imaging specialist not involved in this study. Pathological results were provided by an independent pathologist who was also not involved in this study. The experts conducting imaging and pathological evaluations were blinded to the number of cycles of neoadjuvant immunochemotherapy received by the patients. Cancer types were classified according to the 11th edition of the International Classification of Diseases (ICD-11), with lung adenocarcinoma coded as 2C25.0 and lung squamous cell carcinoma coded as 2C25.28. Pathological complete response (pCR) and MPR were defined based on postoperative pathological examination results. pCR was defined as the absence of viable tumor cells in both the primary tumor and resected lymph nodes, while MPR was defined as residual viable tumor cells comprising no more than 10% of the tissue (2).

All participants who received neoadjuvant therapy underwent CT imaging assessments between the second and third cycles of treatment. The radiological evaluation was based on the Radiological Tumor Size Shrinkage (RTSS) metric (6). The calculation formula is: [(Longest diameter of the primary tumor before treatment - Longest diameter of the primary tumor after treatment)/Longest diameter of the primary tumor before treatment] × 100%. All adverse events were graded and documented according to the National Cancer Institute Common Terminology Criteria for Adverse Events (NCI-CTCAE) version 4.0) (8).

### 2.3 Treatment approach

All participants in the study received 2–3 cycles of neoadjuvant immunochemotherapy prior to surgery. For patients with squamous cell carcinoma, the treatment regimen consisted of platinum-based drugs combined with paclitaxel, docetaxel, or gemcitabine, along with a single immunotherapy agent. For patients with adenocarcinoma, the regimen included platinum-based drugs combined with pemetrexed or paclitaxel. The baseline comparison of the usage of immunotherapy drugs between 2-cycle and 3-cycle is as follows: Sintilimab (19% vs 17%), Camrelizumab (35% vs 41%), Pembrolizumab (36% vs 27%), Tislelizumab (9% vs 10%), Toripalimab (1% vs 1%), Nivolumab (0% vs 1%), Penpulimab (0% vs 1%), Serplulimab (1% vs 0%), Sugemalimab (0% vs 0%) and Adebrelimab (0% vs 0%).

The surgical approach for each patient was determined based on a comprehensive evaluation by the multidisciplinary expert team. Surgical techniques employed included video-assisted thoracoscopic surgery (VATS), robotic-assisted thoracoscopic surgery (RATS), and thoracotomy. All surgical procedures were performed with informed consent from the patients. The extent of resection encompassed wedge (single or multiple lesions), segmentectomy lobectomy (single or multiple lobes), pneumonectomy, with or without sleeve lobectomy, pulmonary artery reconstruction, bronchoplasty, lymph node sampling, or systematic lymph node dissection.

### 2.4 Statistical analysis

This study employed a retrospective cohort design, including data from NSCLC patients who received either two cycles (N = 81) or three cycles (experimental group, N = 229) of neoadjuvant therapy. To address potential confounding in this observational study, we implemented two propensity score (PS) adjustment approaches: IPTW and PSM. The PS was derived from multivariable logistic regression incorporating clinically pertinent covariates (gender, age, smoking history, neoadjuvant treatment regimen, histology, sum of the longest diameters of baseline target lesions, 8th edition cTNM stage). The seven matching factors (e.g., gender, age) minimized selection bias in the study, ensuring baseline balance of key prognostic predictors between the 2-cycle and 3-cycle groups. After matching, the balance of covariates was assessed using the standardized mean difference (SMD), with an SMD < 0.15 indicating effective balance. To verify the stability of the matching, we performed 100 bootstrap iterations to evaluate the distribution of SMDs for each covariate and the fluctuation range of the matched sample size. For IPTW, stabilized inverse probability weights were calculated to estimate the average treatment effect (ATE), with truncation at the 1st and 99th percentiles to mitigate extreme weight influence. Covariate balance was verified using SMD <0.15.

Categorical variables were presented as frequency (percentage), while continuous variables were described using mean ± standard deviation or median (interquartile range) as appropriate. Intergroup comparisons before and after matching were conducted using independent sample t-tests (for normally distributed continuous variables), Mann-Whitney U tests (for non-normally distributed variables), and chi-square tests (for categorical variables). Univariate logistic regression analysis was used to examine the association between the number of neoadjuvant immunochemotherapy cycles and pCR and MPR. COX proportional hazards models were employed to assess the correlation between the number of cycles and overall survival (OS) and disease-free survival (DFS). Kaplan-Meier analysis was used to compare survival differences across different neoadjuvant treatment cycles, MPR, and pCR conditions. Correlation analysis was conducted using univariate logistic regression analysis. Odds Ratio (OR), Hazard ratios (HR) and their associated 95% confidence intervals (95% CI) were calculated. All statistical analyses were performed using R version 4.4.2. *P* < 0.05 indicated statistical significance.

## 3 Results

### 3.1 Baseline characteristics

#### 3.1.1 Comparison of baseline characteristics before PSM and IPW

A total of 310 research subjects were included in the PSM analysis. Prior to PSM, the 2-cycle group consisted of 81 participants with pCR rate of 42.0% (36/81; 95% CI: 31.8%- 52.8%) and MPR rate of 70.4% (57/81; 95% CI: 59.7%-79.2%). 3-cycle group included 229 participants, exhibiting a pCR rate of 40.2% (92/229; 95% CI: 34.0%-46.6%) and an MPR rate of 63.8% (146/229; 95% CI: 57.3%-69.7%). Significant differences were identified between the 3-cycle and 2-cycle groups across several parameters. The 2-cycle group demonstrated a numerically lower Objective Response Rate (ORR)than the 3-cycle group (54% [44/81] vs. 67% [154/229]; *P* = 0.052). Notably, the clinical stage exhibited a significant disparity between the two groups (*P* = 0.011). The interval from the conclusion of neoadjuvant therapy to surgery (OI) was significantly extended in the 3-cycle group compared to the 2-cycle group, with a median of 40 days (IQR: 34-48) versus 35 days (IQR: 30-43) (*P* = 0.002). A higher proportion of subjects with N2 lymph node staging was observed in the 3-cycle group (*P* = 0.047). The subsequent results indicated no statistically significant differences between the groups. The additional results are detailed in **Table 1**. In Treatment-Related Adverse Events (TRAE), the any-grade TRAE rate was 41/81 (49.4%) in the 2-cycle group versus 119/229 (52.0%) in the 3-cycle group (*P* = 0.881; see **Supplementary Table S1**). For Immune-Related Adverse Events (irAEs), any-grade irAEs occurred in 15/81 (18.5%) of the 2-cycle group and 24/229 (10.5%) of the 3-cycle group (*P* = 0.093; see **Supplementary Table S2**).

**Table 1:** Comparison of Baseline Characteristics, PSM, and IPW Data.

Baseline data						PSM						IPW				
Variables	Total (n = 310)	2 (n = 81)	3 (n = 229)	P	SMD	Variables	Total (n = 162)	2 (n = 81)	3 (n = 81)	p	SMD	Variables	2 (n = 79.7)	3 (n = 229.4)	p	SMD
Gender, n (%)				0.709	0.082	Gender, n (%)				1.000	0.049	Gender, n (%)			0.91	0.016
Female	24 (8)	5 (6)	19 (8)			Female	11 (7)	5 (6)	6 (7)			Female	6.6 (8.3)	18.0 (7.8)		
Male	286 (92)	76 (94)	210 (92)			Male	151 (93)	76 (94)	75 (93)			Male	73.1 (91.7)	211.4 (92.2)		
Age, Mean ± SD	60.53 ± 7.69	60.88 ± 7.63	60.41 ± 7.72	0.638	0.061	Age, Mean ± SD	60.94 ± 7.53	60.88 ± 7.63	61.01 ± 7.48	0.909	0.018	Age, Mean ± SD	60.50 (7.53)	60.49 (7.67)	0.988	0.002
Smoke, n (%)				0.478	0.115	Smoke_, n (%)				0.554	0.124	Smoke_, n (%)			0.916	0.015
Never	64 (21)	14 (17)	50 (22)			Never	32 (20)	14 (17)	18 (22)			Never	15.9 (20.0)	47.2 (20.6)		
Current / Former	246 (79)	67 (83)	179 (78)			Current/Former	130 (80)	67 (83)	63 (78)			Current / Former	63.8 (80.0)	182.1 (79.4)		
Histology, n (%)				0.173	0.211	Histology, n (%)				1.000	0.043	Histology, n (%)			0.716	0.054
Adenocarcinoma	47 (15)	8 (10)	39 (17)			Adenocarcinoma	15 (9)	8 (10)	7 (9)			Adenocarcinoma	10.5 (13.1)	34.5 (15.0)		
Squamous cell carcinoma	263 (85)	73 (90)	190 (83)			Squamous cell carcinoma	147 (91)	73 (90)	74 (91)			Squamous cell carcinoma	69.2 (86.9)	194.9 (85.0)		
PTS (mm), Median (Q1, Q3)	49 (40, 64.75)	50 (37, 57)	48 (40, 67)	0.265	0.200	PTS (mm), Median (Q1, Q3)	48 (36.25, 57)	50 (37, 57)	45 (36, 59)	0.809	0.038	PTS (mm), Median (Q1, Q3)	50.87 (16.76)	51.68 (18.65)	0.724	0.046
TLS (mm), Median (Q1, Q3)	56 (45.25, 71)	55 (45, 66)	56 (46, 75)	0.210	0.200	TLS (mm), Median (Q1, Q3)	55 (45, 65)	55 (45, 66)	55 (45, 65)	0.828	0.049	TLS (mm), Median (Q1, Q3)	58.98 (16.73)	59.71 (19.26)	0.753	0.041
Clinical Stage, n (%)				0.011	0.417	Clinical Stage, n (%)				0.990	0.053	Clinical Stage, n (%)			0.987	0.049
IIA	24 (8)	5 (6)	19 (8)			IIA	10 (6)	5 (6)	5 (6)			IIA	7.3 (9.1)	18.0 (7.8)		
IIB	73 (24)	30 (37)	43 (19)			IIB	59 (36)	30 (37)	29 (36)			IIB	19.1 (24.0)	54.2 (23.6)		
IIIA	132 (43)	29 (36)	103 (45)			IIIA	60 (37)	29 (36)	31 (38)			IIIA	33.1 (41.5)	97.5 (42.5)		
IIIB	81 (26)	17 (21)	64 (28)			IIIB	33 (20)	17 (21)	16 (20)			IIIB	20.2 (25.4)	59.8 (26.1)		
Node stage, n (%)				0.066	0.299	Node stage, n(%)				0.676	0.139	Node stage, n(%)			0.957	0.040
N0	58(19)	16(20)	42(18)			N0	35(22)	16(20)	19(23)			N0	15.7(19.7)	42.7(18.6)		
N1	83(27)	29(36)	54(24)			N1	53(33)	29(36)	24(30)			N1	21.7(27.2)	60.6(26.4)		
N2	169(55)	36(44)	133(58)			N2	74(46)	36(44)	38(47)			N2	42.3(53.0)	126.0(54.9)		
N2 stage, n (%)				0.047	0.275	N2 stage,n (%)				0.875	0.049	N2 stage,n (%)			0.776	0.038
No	141(45)	45(56)	96(42)			No	88(54)	45(56)	43(53)			No	37.4(47)	103.3(45.1)		
Yes	169(55)	36(44)	133(58)			Yes	74(46)	36(44)	38(47)			Yes	42.3(53)	126.0(54.9)		
RTSS, Mean ± SD	30.47 ± 22.65	30.02 ± 20.7	30.63 ± 23.35	0.826	0.028	RTSS, Mean ± SD	31.02 ± 22.33	30.02 ± 20.7	32.03 ± 23.93	0.568	0.090	RTSS, Mean ± SD	29.90 (20.14)	30.69 (23.36)	0.775	0.036
RTSS_2vs3, Mean ± SD	37.16 ± 23.13	30.02 ± 20.7	39.69 ± 23.46	< 0.001	0.437	RTSS_2vs3, Mean ± SD	34.93 ± 24.33	30.02 ± 20.7	39.85 ± 26.71	0.010	0.412	RTSS_2vs3, Mean ± SD	29.90 (20.14)	39.74 (23.66)	<0.001	0.448
ORR,n (%)				0.052	0.267	ORR,n (%)				0.525	0.125	ORR			0.078	0.238
No	112 (36)	37 (46)	75 (33)			No	69 (43)	37 (46)	32 (40)			No	35.6 (44.6)	76.0 (33.1)		
Yes	198 (64)	44 (54)	154 (67)			Yes	93 (57)	44 (54)	49 (60)			Yes	44.1 (55.4)	153.4 (66.9)		
NT, n (%)				0.365	0.231	NT, n (%)				0.981	0.066	NT, n (%)			0.979	0.059
Camrelizumab	122 (39)	28 (35)	94 (41)			Camrelizumab	57 (35)	28 (35)	29 (36)			Camrelizumab	30.4 (38.2)	89.8 (39.2)		
other	44 (14)	9 (11)	35 (15)			other	18 (11)	9 (11)	9 (11)			other	9.9 (12.5)	32.1 (14.0)		
Pembrolizumab	90 (29)	29 (36)	61 (27)			Pembrolizumab	59 (36)	29 (36)	30 (37)			Pembrolizumab	24.8 (31.1)	67.1 (29.2)		
Sintilimab	54 (17)	15 (19)	39 (17)			Sintilimab	28 (17)	15 (19)	13 (16)			Sintilimab	14.5 (18.2)	40.3 (17.6)		
OI (days), Median (Q1, Q3)	39 (33, 47)	35 (30, 43)	40 (34, 48)	0.002	0.320	OI (days), Median (Q1, Q3)	36.5 (31, 46)	35 (30, 43)	39 (33, 48)	0.034	0.343	OI (days), Median (Q1, Q3)	38.14 (12.21)	43.59 (19.06)	0.004	0.341
Change, n (%)				0.140	0.195	Change, n (%)				0.167	0.272	Change, n (%)			0.106	0.194
No	294 (95)	74 (91)	220 (96)			No	153 (94)	74 (91)	79 (98)			No	73.0 (91.6)	220.6 (96.2)		
Yes	16 (5)	7 (9)	9 (4)			Yes	9 (6)	7 (9)	2 (2)			Yes	6.7 (8.4)	8.7 (3.8)		
OT (mins), Median (Q1, Q3)	150 (120, 194.25)	140 (120, 195)	155 (120, 192)	0.470	0.092	OT (mins), Median (Q1, Q3)	150 (120, 191.5)	140 (120, 195)	160 (127, 190)	0.394	0.124	OT (mins), Median (Q1, Q3)	161.85 (53.54)	164.99 (56.43)	0.666	0.057
Surgery, n (%)				0.494	0.180	Surgery, n (%)				0.577	0.227	Surgery, n (%)			0.495	0.18
Lobectomy	275 (89)	70 (86)	205 (90)			Lobectomy	139 (86)	70 (86)	69 (85)			Lobectomy	69.6 (87.3)	205.8 (89.7)		
Pneumonectomy	33 (11)	11 (14)	22 (10)			Pneumonectomy	21 (13)	11 (14)	10 (12)			Pneumonectomy	10.1 (12.7)	21.2 (9.2)		
Segmentectomy	2 (1)	0 (0)	2 (1)			Segmentectomy	2 (1)	0 (0)	2 (2)			Segmentectomy	0.0 (0.0)	2.4 (1.0)		
Blood (ml), Median (Q1, Q3)	150 (100, 200)	200 (100, 300)	150 (100, 200)	0.191	0.137	Blood (ml), Median (Q1, Q3)	150 (100, 245)	200 (100, 300)	150 (100, 200)	0.385	0.067	Blood (ml), Median (Q1, Q3)	234.04 (246.35)	213.08 (274.27)	0.506	0.08
Complication, n (%)				0.809	0.062	Complication, n (%)				1.000	0.045	Complication, n (%)			0.737	0.042
No	287 (93)	74 (91)	213 (93)			No	149 (92)	74 (91)	75 (93)			No	73.6 (92.3)	214.2 (93.4)		
Yes	23 (7)	7 (9)	16 (7)			Yes	13 (8)	7 (9)	6 (7)			Yes	6.1 (7.7)	15.2 (6.6)		
Pulmonary complications, n (%)				0.220	0.174	Pulmonary complications, n (%)				0.209	0.225	Pulmonary complications, n (%)			0.508	0.086
No	232(75)	56(69)	176(77)			No	120(74)	56(69)	64(79)			No	58.3(73.1)	176.1(76.8)		
Yes	78(25)	25(31)	53(23)			Yes	42(26)	25(31)	17(21)			Yes	21.5(26.9)	53.3(23.2)		
CDTD (days), Median (Q1, Q3)	7 (5, 9)	7 (5, 9)	6 (5, 9)	0.235	0.015	CDTD (days), Median (Q1, Q3)	6 (5, 9)	7 (5, 9)	6 (4, 9)	0.127	0.020	CDTD (days), Median (Q1, Q3)	7.43 (3.42)	7.57 (4.88)	0.775	0.034
HD (days), Median (Q1, Q3)	10 (7, 12)	10 (8, 13)	9 (7, 12)	0.053	0.118	HD (days), Median (Q1, Q3)	10 (7, 13)	10 (8, 13)	9 (7, 13)	0.104	0.091	HD (days), Median (Q1, Q3)	10.53 (3.62)	10.26 (4.90)	0.607	0.063
pCR, n (%)				0.879	0.037	pCR, n (%)				0.528	0.124	pCR, n (%)			0.801	0.034
Neg	184 (59)	47 (58)	137 (60)			Neg	89 (55)	47 (58)	42 (52)			Neg	44.9 (56.3)	133.0 (58.0)		
Pos	126 (41)	34 (42)	92 (40)			Pos	73 (45)	34 (42)	39 (48)			Pos	34.8 (43.7)	96.3 (42.0)		
MPR, n (%)				0.347	0.141	MPR, n (%)				0.735	0.080	MPR, n (%)			0.44	0.106
Neg	107 (35)	24 (30)	83 (36)			Neg	51 (31)	24 (30)	27 (33)			Neg	24.3 (30.5)	81.3 (35.4)		
Pos	203 (65)	57 (70)	146 (64)			Pos	111 (69)	57 (70)	54 (67)			Pos	55.4 (69.5)	148.1 (64.6)		

PSM, Propensity Score Matching; IPW, Inverse Probability Weighting; PTS, Primary Tumor Size; TLS, Target Lesion Size (Based on RECIST 1.1 Criteria); T stage, The T staging in the 8th edition of AJCC clinical TNM staging for non-small cell lung cancer (NSCLC); Node stage, The N staging in the 8th edition of AJCC clinical TNM staging for NSCLC; N2 stage, Instances of N2 staging in N classification; RTSS, Radiological tumor size shrinkage; ORR, he Objective Response Rate , which includes Complete Response(Disappearance of all target lesions and no new lesions) and Partial Response(≥30% decrease in sum of target lesion diameters). NT, The immunotherapy agent of Neoadjuvant Therapy; Other, Including Tislelizumab, Toripalimab, Nivolumab, Penpulimab, Serplulimab, Sugemalimab and Adebrelimab. OI, The time interval from the end of the last cycle of neoadjuvant therapy to the surgery; Change, The situation of conversion to thoracotomy during the operation; OT, Operation time; Blood, Intraoperative blood loss volume; Complications, Post-operative complications(Including electrolyte imbalance, pleural effusion, pneumothorax, bronchopleural fistula, arrhythmias, and infections); Pulmonary complications, Including postoperative atelectasis, pulmonary infection, atelectasis and chylothorax. CDTD, The duration of chest drainage tube placement; HD, Duration of hospitalization; pCR, Pathological complete response; MPR, Major pathological response; NEG, No pCR achievement; POS, Achieve pCR.

#### 3.1.2 Evaluation of PSM and IPW

The matched variables included gender, age, smoking history, neoadjuvant treatment regimen, histology subtype stratification (squamous cell carcinoma and adenocarcinoma), sum of the longest diameters of baseline target lesions, 8th edition cTNM stage. After PSM, the covariates and N stage were well-balanced across the groups. There were no statistically significant differences in the covariates. SMDs for all covariates were below 0.15, demonstrating excellent balance. After 100 bootstrap iterations, our analysis revealed that the distribution of standardized mean differences (SMDs) for each covariate, along with the variability in the matched sample size, indicated positive outcomes (**Supplementary Figure S1**). This outcome is indicative of a robust balance achievement between the compared groups, suggesting that our matching procedure was successful in reducing observable disparities between treatment and control cohorts. After PSM, the baseline data between the groups were well matched, with 81 subjects in each of the 2-cycle and 3-cycle groups. The detailed comparison results are shown in **Table 1**. After IPW, the baseline data between the groups were well matched with all SMDs less than 0.15(**Supplementary Figure S2**). Weight-adjusted characteristics were documented in **Table 1**.

#### 3.1.3 Correlation analysis between RTSS and pathological responses (pCR/MPR)

Logistic regression analysis demonstrated a significant positive association between RTSS and pCR positivity (OR = 1.02, 95% CI [1.01–1.03], p = 0.002), indicating that a 1% increase in RTSS is associated with approximately a 2% higher likelihood of achieving pCR. Furthermore, RTSS was significantly associated with MPR positivity (OR = 1.03, 95% CI [1.01–1.04], p < 0.001), with each 1% increment in RTSS linked to an approximate 3% increase in the probability of MPR.

### 3.2 Comparison of 2-cycle and 3-cycle results

#### 3.2.1 Comparison of short-term efficacy

##### 3.2.1.1 Comparison of pathological remission indicators(pCR and MPR)

In terms of pathological remission, the comparison results between the two groups after PSM and IPW were consistent. After IPW, the pCR rate was 42.0% (95% CI: 35.6-48.7%) after 3-cycle and 43.7% (95% CI: 32.5-55.5%) after 2-cycle (odds ratio (OR), 0.933; 95%CI: 0.542-1.604, *P* = 0.801). The MPR rate was 64.6% (95% CI: 58.1-70.6%) after 3-cycle and 69.5% (95%CI: 57.8%-79.2%) after 2-cycle (OR, 0.798; 95%CI: 0.449-1.418, *P* = 0.440).

**Figure 2:**
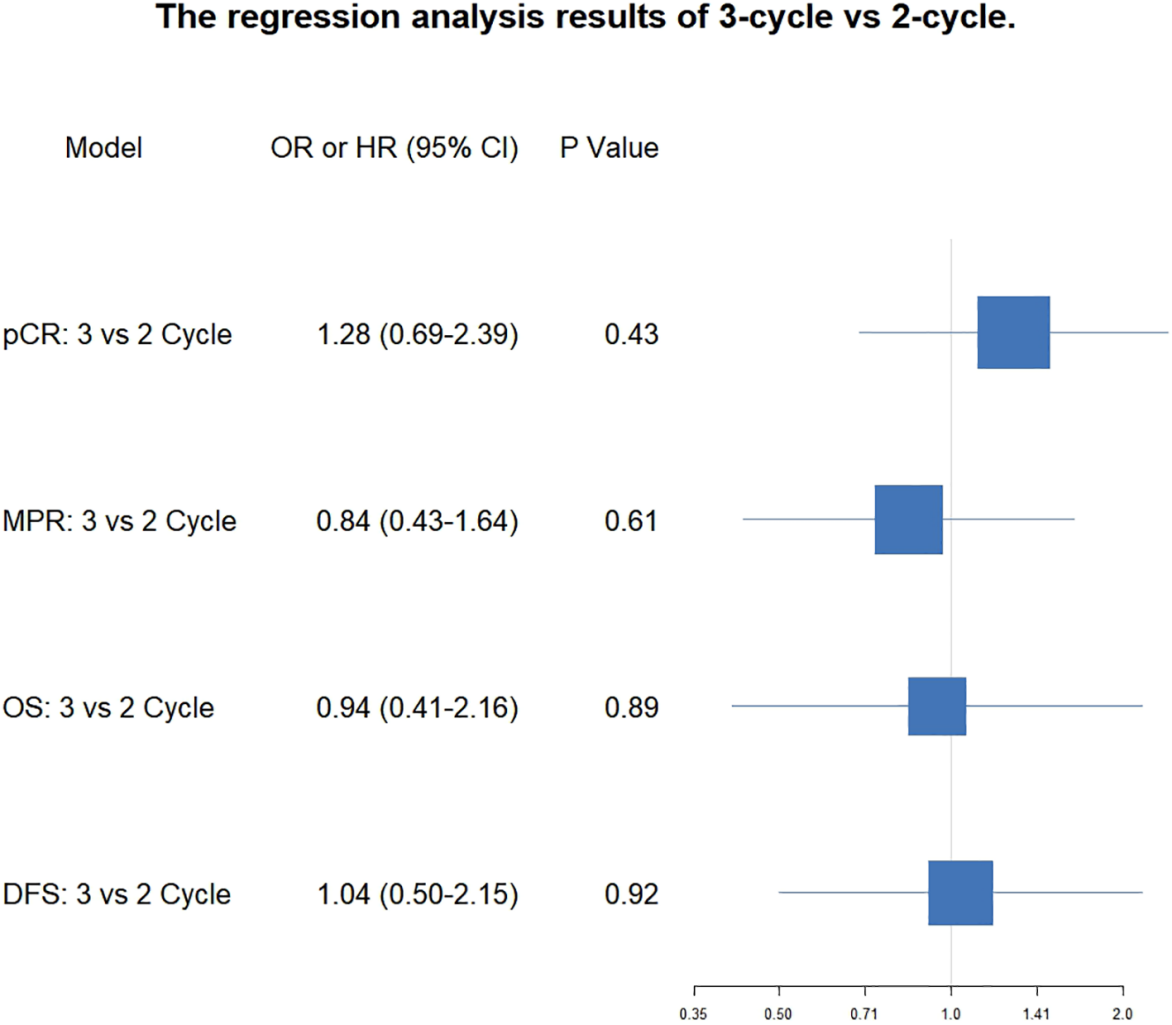
Forest plot of the correlation between the number of treatment cycles and pCR, MPR, OS and DFS.

After PSM, the pCR rate was 48.1% (39/81, 95% CI: 37.6-58.9%) after 3-cycle and 42.0% (34/81, 95% CI: 31.8-52.8%) after 2-cycle (OR, 1.284; 95%CI: 0.691-2.395, *P* = 0.430). The MPR rate was 66.7% (54/81, 95% CI: 55.9-76.0%) after 3-cycle and 70.4% (57/81, 95%CI: 59.7%-79.2%) after 2-cycle (OR, 0.842; 95%CI: 0.432-1.636, *P* = 0.612). The analysis revealed that the extension of neoadjuvant immunochemotherapy from two to three cycles did not yield a statistically significant improvement in pCR or MPR rates. The proportion of patients attaining pCR or MPR remained consistent across both treatment durations, suggesting that the therapeutic efficacy in terms of pathological response is equivalent between the 2-cycle and 3-cycle regimens. The forest plot for the correlation analysis between treatment cycles and pathological remission status (pCR and MPR) is shown in **Figure 2**.

We conducted additional subgroup analyses. First, by comparing pathological responses between the 2-cycle and 3-cycle groups across clinical stage subgroups (IIA-IIIB) at baseline and post-PSM, we observed no statistically significant differences; however, post-PSM analysis revealed pCR rates of 100% (3-cycle) vs 60.0% (2-cycle) (*P* = 0.444) and MPR rates of 100% vs 100% (*P* = 1.000) in stage IIA patients, suggesting that three cycles of neoadjuvant immunochemotherapy may facilitate pCR achievement in this subgroup (**Supplementary Figure S3**). Second, comparisons between groups stratified by target lesion size (TLS) (≥5cm vs <5cm) at baseline and post-PSM demonstrated no significant intergroup differences (**Supplementary Figure S4**).

##### 3.2.1.2 Comparison of perioperative safety

Following PSM, the incidence of TRAEs was identical between groups. Any-grade TRAEs occurred in 41/81 patients (49.4%) in both the 2-cycle and 3-cycle groups (*P* = 1.000; **Supplementary Table S1**). For irAEs, any-grade irAEs occurred in 15/81 patients (18.5%) receiving 2 cycles compared to 8/81 patients (9.9%) receiving 3 cycles (*P* = 0.177; **Supplementary Table S2**). In terms of perioperative safety, a notable difference in OI was observed between the 3-cycle and 2-cycle regimens, a finding that remained consistent across both PSM and IPW. The 3-cycle regimen necessitated a longer interval than the 2-cycle regimen, as evidenced by a median duration of 39.0 days (interquartile range: 33.0 to 48.0 days) compared to 35.0 days (interquartile range: 30.0 to 43.0 days) following PSM, with a statistically significant difference (*P* = 0.034). Similarly, after IPW, the mean duration was 43.59 ± 19.06 days for the 3-cycle regimen versus 38.14 ± 12.21 days for the 2-cycle regimen, also demonstrating statistical significance (*P* = 0.004). In addition, no significant differences were observed between the 2-cycle and 3-cycle groups in terms of hospitalization duration (HD) [3-cycle vs. 2-cycle, 9.0 (7.0, 13.0) days vs. 10.0 (8.0, 13.0) days, *P* = 0.104] after PSM and [3-cycle vs. 2-cycle, 10.3 ± 4.9 days vs. 10.5 ± 3.6 days, *P* = 0.607] after IPW, the duration of chest drainage tube placement (CDTD) [3-cycle vs. 2-cycle, 6.0(4.0, 9.0) days vs. 7.0 (5.0, 9.0) days, *P* = 0.127] after PSM and [3-cycle vs. 2-cycle, 7.6 ± 4.9 days vs. 7.4 ± 3.4 days, *P* = 0.775] after IPW, operation time [3-cycle vs. 2-cycle, 160 (127, 190) mins vs. 140.0 (120.0, 195.0) mins, *P* = 0.394] after PSM and [3-cycle vs. 2-cycle, 165.0 ± 56.4mins vs. 161.9 ± 53.5 mins, *P* = 0.666] after IPW, surgical blood loss [3-cycle vs. 2-cycle, 150.0 (100.0, 200.0) ml vs. 200.0 (100.0, 300.0) ml, *P* = 0.385] after PSM and [3-cycle vs. 2-cycle, 213.1 ± 274.3 ml vs. 234.0 ± 246.4 ml, *P* = 0.506] after IPW, conversion to thoracotomy during the operation [3-cycle vs. 2-cycle, 2/81 (2.4%) vs. 7/81 (8.6%), *P* = 0.167] after PSM and [3-cycle vs. 2-cycle, 8.7/229.4 (3.8%) vs. 6.7/79.7 (8.4%), *P* = 0.106] after IPW, postoperative complication rates [3-cycle vs. 2-cycle, 6/81 (7.4%) vs. 7/81 (8.6%), *P* = 1.000] after PSM and [3-cycle vs. 2-cycle, 15.2/229.4 (6.6%) vs. 6.1/79.7 (7.7%), *P* = 0.737] after IPW. Pulmonary complications rates [3-cycle vs. 2-cycle, 17/81 (21%) vs. 25/81 (31%), *P* = 0.209] after PSM and [3-cycle vs. 2-cycle, 23.2%) vs. 26.9%), *P* = 0508] after IPW. The results are presented in **Table 1** for comparison.

### 3.3 Comparison of survival outcomes

The median follow-up time for the 3-cycle group was 25.3months, and the median DFS for the 3-cycle group was not reached (95% CI: NR-NR). The median follow-up time for the 2-cycle group was 31.3 months, and the median DFS for the 2-cycle group was also not reached (95% CI: NR-NR). The comparison between the 3-cycle and 2-cycle groups showed no significant difference in DFS (Hazard Ratio (HR) = 1.04 [0.50-2.15], *P* = 0.921). The three-year DFS rate for the three-cycle regimen was 84.6% (95% CI: 77.1% - 89.8%), whereas the two-cycle regimen exhibited a three-year DFS rate of 88.2% (95% CI: 77.5% - 94.0%). Statistical analysis indicated that there was no significant difference in the three-year DFS rates between the two regimens (*P* = 0.921). The results indicate that, within the duration of the current follow-up period, there was no significant difference in DFS between patients undergoing two versus three cycles of neoadjuvant therapy.

Regarding OS, the median OS for the 3-cycle group was not reached (95% CI: NR-NR), and the median OS for the 2-cycle group was also not reached (95% CI: NR-NR). The comparison of OS between the two groups showed no significant difference (HR = 0.94 [0.41-2.16], *P* = 0.892). The three-year OS rate for the three-cycle regimen was 88.6% (95% CI: 81.5% - 93.1%), whereas the two-cycle regimen exhibited a three-year OS rate of 88.2% (95% CI: 77.5% - 94.0%). Statistical analysis indicated that there was no significant difference in the three-year DFS rates between the two regimens (*P* = 0.892). The results suggest that, within the current follow-up period, there is no significant difference in OS between patients receiving two cycles versus three cycles of neoadjuvant therapy. The forest plot illustrating the correlation analysis between the number of treatment cycles and survival outcomes, including both overall survival OS and DFS, is presented in **Figure 2**.

The study indicated that MPR and pCR possess prognostic significance for DFS and OS. In the DFS analysis, the median survival was not reached in the MPR group (95% CI: NR-NR), while the non-MPR group was 49.3(95% CI: 45.9-NR), with a statistically significant hazard ratio of 0.25 (95% CI: 0.13-0.50; *P*<0.001). The three-year DFS rate was 91.8% (95% CI: 85.1%-95.5%) in the MPR group, compared to 74.6% (95% CI: 62.5%-83.2%) in the non-MPR group. Regarding OS, the median survival was not reached in the MPR group (95% CI: NR-NR), while the non-MPR group also did not reach median survival (95% CI: 49.3-NR), with a statistically significant hazard ratio of 0.30 (95% CI: 0.14-0.65; *P* = 0.002). The three-year OS rate for the MPR group was 93.3% (95% CI: 86.9%-96.6%), in contrast to 79.6% (95% CI: 67.5%-87.5%) in the non-MPR group. The median follow-up durations were 26.2 months for MPR patients and 26.9 months for non-MPR patients. For detailed survival data pertaining to various groups, please consult **Table 2**.

**Table 2:** Survival times (DFS and OS) for different treatment cycles and pathological remission statuses.

		Median OS (95% CI) (months)	Median DFS (95% CI) (months)	Median Follow up(months)
Cycle	2	NR(NR-NR)	NR (NR-NR)	31.3
	3	NR (NR-NR)	NR (NR-NR)	25.3
pCR	NEG	NR (NR-NR)	NR (NR-NR)	28.8
	POS	NR(NR-NR)	NR(NR-NR)	25.5
MPR	NEG	NR (49.3-NR)	49.3 (45.9-NR)	26.9
	POS	NR(NR-NR)	NR(NR-NR)	26.2

Cycle, The number of neoadjuvant therapy cycles; pCR, Pathological complete response; MPR, Major pathological response; NEG, No pCR or MPR achievement; POS, Achieve pCR or MPR; NR, Not Reached.

**Figure 3:**
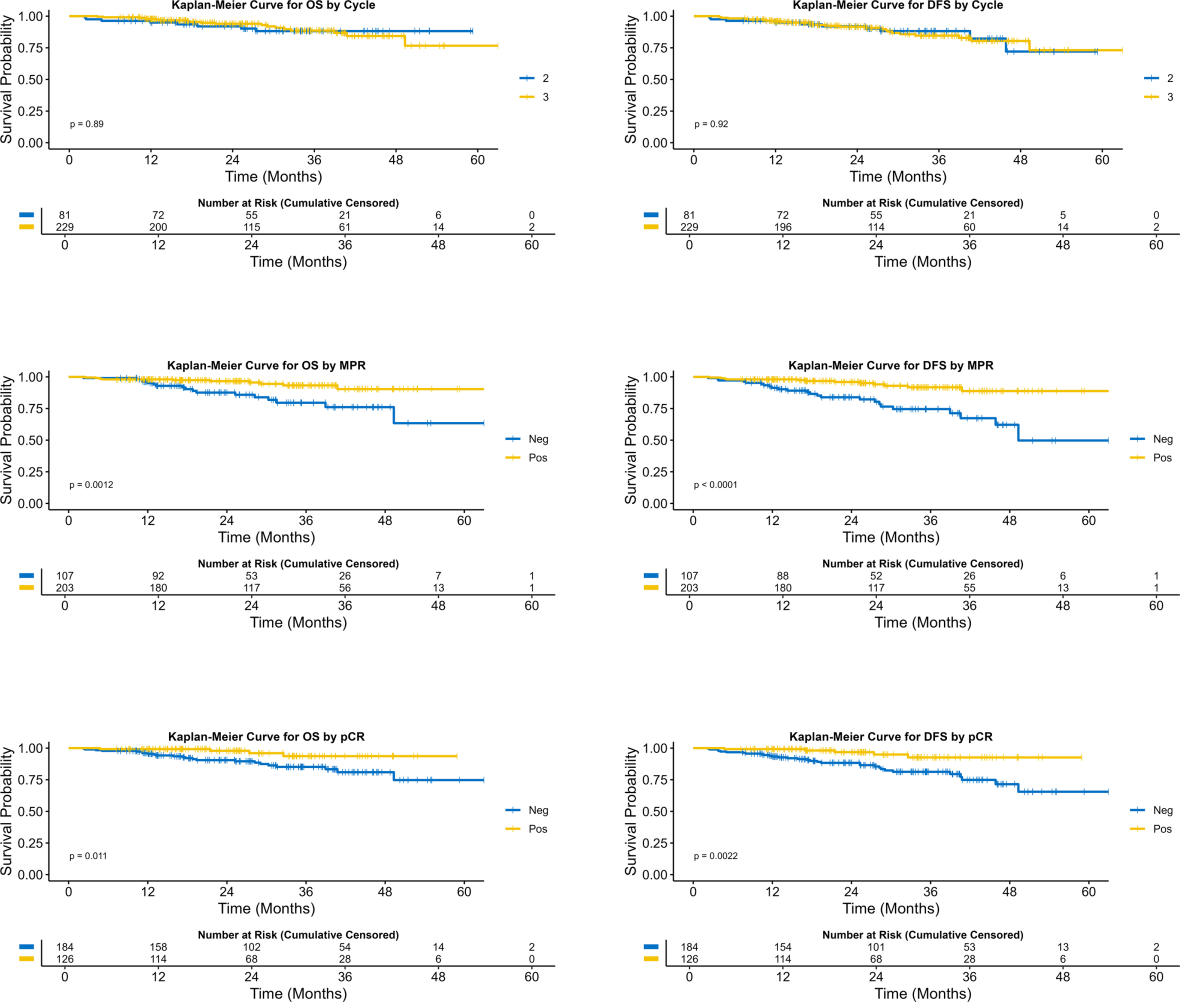
Comparison of survival curves across different neoadjuvant treatment cycles, MPR status, and pCR status.

Moreover, the median disease-free survival (DFS) was not attained in either the pCR or non-pCR cohorts, with the 95% CI extending from NR to NR. The hazard ratio was 0.25 (95% CI: 0.10-0.65; *P* = 0.005), indicating significant difference. The three-year DFS rate was 92.6% (95% CI: 82.1%-97.1%) for the pCR group and 81.3% (95% CI: 73.3%-87.1%) for the non-pCR group. In terms of OS, neither group achieved a median survival, with a 95% CI of NR to NR, and the difference between groups was statistically significant (HR: 0.28; 95% CI: 0.10-0.80; *P* = 0.018). The three-year OS rate was 93.6% (95% CI: 82.9%-97.7%) for the pCR group, compared to 85.1% (95% CI: 77.5%-90.4%) for the non-pCR group. The median follow-up durations were 25.5 months for patients in the pCR group and 28.8 months for those in the non-pCR group. **Figure 3** illustrates the Kaplan-Meier curves for the various groups.

## 4 Discussion

Neoadjuvant therapy is now frequently used for NSCLC (2, 9–13). This approach was classically defined as systemic treatment administered before local therapy. Potential advantages of neoadjuvant therapy include: providing a favorable microenvironment for immune responses, achieving better treatment compliance, enabling direct assessment of therapeutic efficacy, facilitating minimally invasive surgery for organ preservation through tumor downstaging, and improving both distant and local control (14, 15). However, disadvantages encompass potential delays in initiating definitive treatment, increased treatment toxicity, challenges in accurate pathological staging, and elevated surgical complexity compared to patients not receiving neoadjuvant therapy (14–16).

Given the potential disadvantage of neoadjuvant therapy delaying definitive surgery, the selection of treatment cycles becomes a critical clinical decision requiring meticulous consideration. Based on the significant efficacy of neoadjuvant immunochemotherapy demonstrated in clinical trials such as CheckMate 816 (2) and CheckMate 77T (13) the NCCN now recommends a 4-cycle neoadjuvant treatment regimen for NSCLC patients who meet preoperative criteria (17). Although the efficacy of neoadjuvant therapy using 3 or 4 cycles has been validated by numerous Phase III clinical trials (2, 13, 18–20), uniformly applying a 4-cycle neoadjuvant regimen to all NSCLC patients does not align with the current trend toward individualized treatment. Furthermore, numerous clinical trials of preoperative single-agent immunotherapy have opted for two-cycle treatment regimens (4, 5, 21). In clinical trials, it is essential to ensure that patients in both the control and experimental groups undergo the same number of treatment cycles to achieve a scientifically valid evaluation of therapeutic efficacy. However, the current selection of neoadjuvant treatment cycle numbers appears to be influenced by the personal experience of trial investigators and their confidence in the chosen treatment regimen. The efficacy of neoadjuvant immunochemotherapy in NSCLC has been remarkable. Therefore, the selection of treatment cycle numbers must be approached with greater caution and supported by robust evidence demonstrating the differences between various cycle regimens and their impacts on different patient populations. This will help maximize the benefits of neoadjuvant therapy for NSCLC patients while striving to minimize or even prevent adverse outcomes associated with this treatment.

neoSCORE, as the first known clinical trial to investigate the differences in neoadjuvant treatment cycle numbers, provides invaluable insights for optimizing the selection of treatment cycles (6). In this study, Miner Shao et al. reported that compared with two cycles of neoadjuvant therapy, three cycles resulted in a higher MPR rate (41.4% vs 26.9%; *P* = 0.260). Numerically, the three-cycle neoadjuvant chemotherapy-immunotherapy regimen appeared more favorable for achieving MPR. However, the data safety monitoring board (DSMB) halted the trial after enrolling 60 participants, as it determined that the MPR rate in the three-cycle group was unlikely to show significant superiority over the two-cycle group (6).

We must acknowledge the significant contribution and pivotal role of the neoScore study in informing the selection of neoadjuvant treatment cycles. However, given the insights provided by the neoScore results, additional reports are warranted to further investigate this issue until a definitive conclusion can be reached. In this study, we compared the MPR rate, pCR rate, OS, and DFS between the 2-cycle and 3-cycle regimens. However, no statistically significant differences were observed across these endpoints. From a quantitative standpoint, it is noteworthy that although the pathological complete response (pCR) rate was elevated in the 3-cycle group relative to the 2-cycle group, the major pathological response (MPR) rate was diminished in the 3-cycle group compared to the 2-cycle group. The observed inconsistency in the numerical results may be ascribed to random variations, which could also suggest that the number of neoadjuvant treatment cycles does not exert a significant influence on pathological remission.

There is difference in Major Pathological Response (MPR) rates between our study and the neoSCORE trial. In the neoSCORE trial, the 3-cycle vs 2-cycle MPR rates were 41.4% (12/29) vs 26.9% (7/26), while our post-PSM results showed 70.4% (57/81) vs 66.7% (54/81). However, nearly all Relative Risk (RR) values >1 in the neoSCORE trial baseline comparisons (except stage IIIA) indicate the 3-cycle group had higher proportions of males, elevated PD-L1 expression, more stage III patients, more squamous histology cases, and more smokers (numerically higher but statistically non-significant) (6). Among these factors, trials including RATIONALLE-315 (22), AEGEAN (23), CheckMate-77T (13), and CheckMate-816 (2) demonstrate superior pathological response benefits in squamous NSCLC patients; higher PD-L1 expression correlates with improved outcomes (24); Stage III patients show enhanced response to neoadjuvant immunochemotherapy (2, 13); smoking history associates with immunotherapy benefit (25, 26); and males derive greater immunotherapy benefit than females (27). Thus, the neoSCORE trial’s 3-cycle group—enriched with high PD-L1 expressors, squamous histology, stage III patients, and smokers—may have contributed to outcome differences. In contrast, our study achieved near-perfect balance in these treatment-influencing confounders through PSM and IPW, minimizing confounding effects. This likely explains our study’s more comparable MPR rates. Additionally, our cohort received a variety of PD-1/PD-L1 inhibitors (e.g., Pembrolizumab, Nivolumab, Atezolizumab, Toripalimab, Camrelizumab, Sintilimab). This heterogeneity reflects real-world practice but introduces a variable not present in the single-agent neoSCORE trial (6). It is possible that the overall efficacy profile across this diverse group might differ slightly from the focused use of Sintilimab in a highly responsive tumor type. Finally, neoSCORE (6) utilize stringent eligibility criteria to enroll a homogenous cohort with defined disease stages, optimal performance status, preserved organ function, and limited comorbidities. This design enhances internal validity and identifies efficacy in an optimized population. Conversely, our real-world study intentionally captured the inherent heterogeneity of routine clinical practice. By including patients with diverse comorbidities, varying performance status, and broader disease characteristics, our findings reflect the “effectiveness” of the intervention across the spectrum of patients encountered by oncologists, thereby enhancing external validity and generalizability.

Our finding that there was no significant difference in pathological response (pCR/MPR) between the 2-cycle and 3-cycle groups suggests a potential mechanistic plateau in immune activation. We hypothesize that the first two cycles of chemotherapy, by inducing immunogenic cell death, creates a pro-inflammatory microenvironment and rapidly expands the pool of tumor-reactive T cells, as evidenced by the significant early surge in tumor-infiltrating lymphocytes (TILs) and CD8+ T cell proportions observed after just one cycle of neoadjuvant chemotherapy (NAC) (28). Concurrent checkpoint blockade is critical in this phase, likely acting to sustain the proliferation and effector differentiation of pre-existing PD-1– CD8+ TILs, particularly the *Tcf7*+ memory-precursor-like subset, which is essential for an effective anti-tumor response and expands upon immunotherapy (29). We posit that this first-two-cycle synergy achieves a maximal practical recruitment and activation of the available anti-tumor T cell repertoire. Consequently, a third cycle provides diminishing returns, as it may fail to mobilize new, potent T cell clones. Instead, persistent antigen exposure and inflammatory signals could push the early-activated T cells toward exhaustion, while the residual tumor microenvironment evolves toward a more immunosuppressive state, characterized by a decline in TIL density and an increase in immunosuppressive M2 macrophages at the end of treatment, as documented in NAC non-responders (28). Thus, the lack of additional benefit from one-cycle extension may stem from an inability to further amplify the cytotoxic immune response, coupled with the inadvertent promotion of T cell dysfunction and compensatory immunosuppression.

Regarding perioperative safety, our findings suggest that patients who received two cycles of neoadjuvant therapy experienced more favorable outcomes during the perioperative period compared to those who underwent three cycles. The extended operative interval (OI) associated with the three-cycle regimen may be attributed to the additional recovery time required following the third cycle of immunochemotherapy. This prolonged interval could potentially affect treatment schedules and resource allocation in clinical settings. Furthermore, when comparing other perioperative outcomes, increasing the neoadjuvant treatment from two to three cycles did not confer any advantages in perioperative safety indicators, indicating that the perioperative safety of the two regimens is approximately equivalent.

While precise quantification of surgery ineligibility per cycle remains elusive in retrospective cohorts, our integrative analysis of trial data and clinician consensus suggests marginally higher attrition after 2 cycles (12%) versus 3 cycles (10%). This underscores the need for prospective recording of discontinuation drivers in future studies. Furthermore, given the real-world nature of this study, adverse event (AE) monitoring was subject to delayed assessments, potentially compromising both AE grading accuracy and detection sensitivity. These limitations may have resulted in false-negative AE reporting for some patients. More robust data from prospective cohort studies are needed to further elucidate AE incidence patterns.

In conclusion, Our analysis demonstrated comparable pathological responses (pCR/MPR) and survival outcomes (DFS/OS) between 2-cycle and 3-cycle neoadjuvant immunochemotherapy, while the two-cycle regimen was associated with a significantly shorter neoadjuvant-to-surgery interval, potentially facilitating earlier surgical intervention.

## Data Availability

The original contributions presented in the study are included in the article/**Supplementary Material**. Further inquiries can be directed to the corresponding authors.
