# Supplementary figures and images for: Two vs three cycles of neoadjuvant immunochemotherapy for resectable non-small-cell lung cancer: a real-world population-based study
Source: Front Immunol. 2025 Nov 17;16:1654830. doi: 10.3389/fimmu.2025.1654830 (PMC12665730; doi:10.3389/fimmu.2025.1654830)

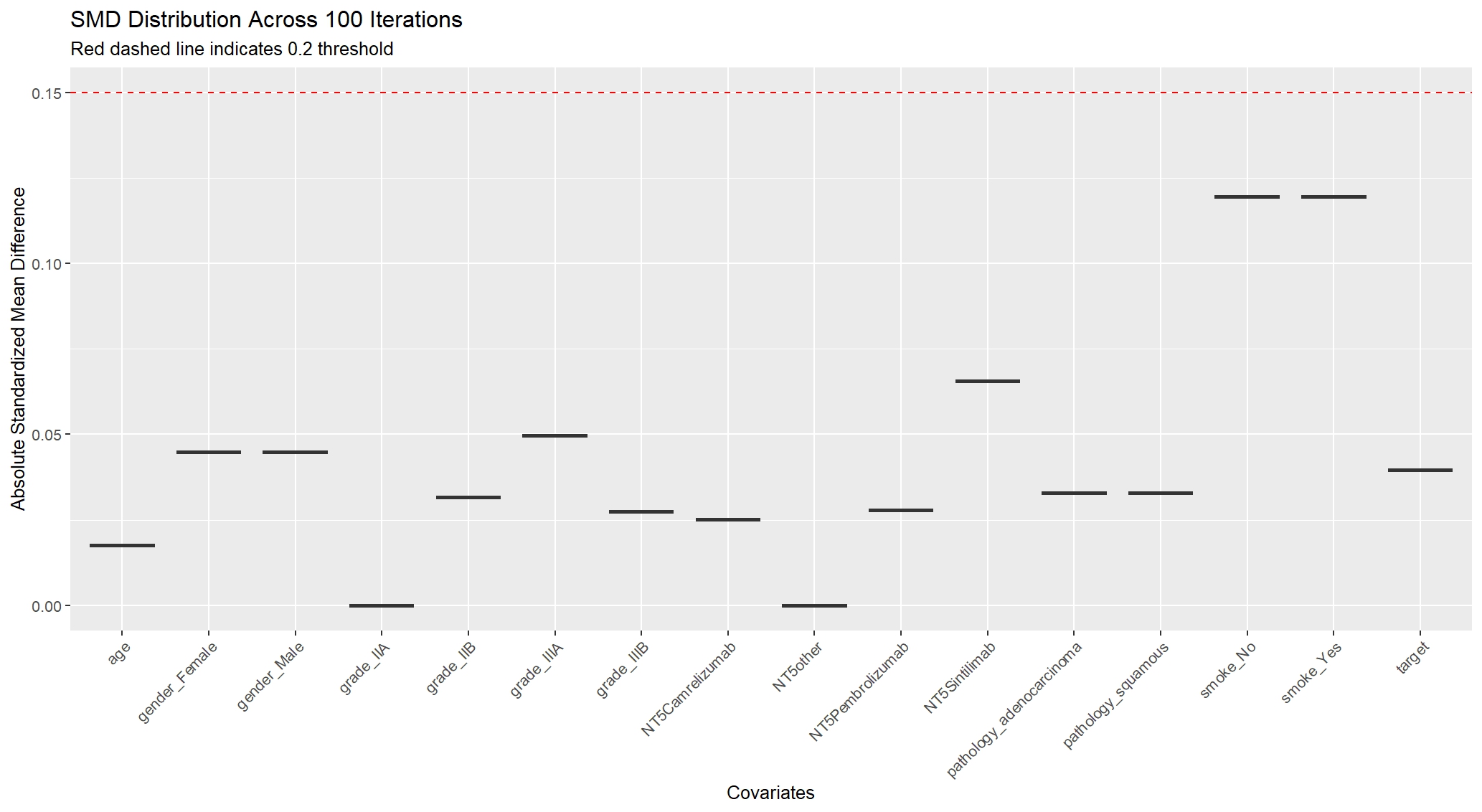

Supplement: Supplementary file 1 [file Image1.jpeg]

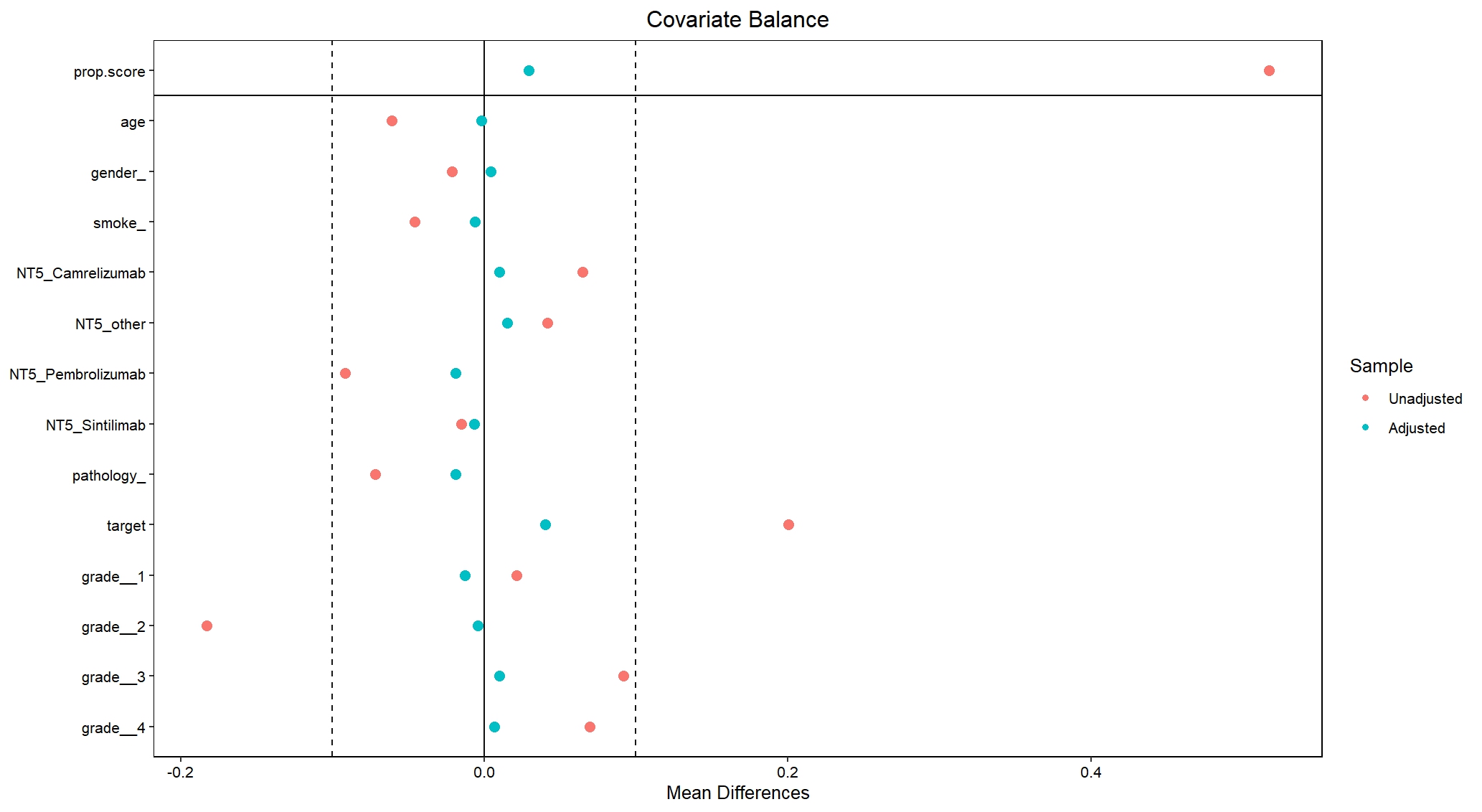

Supplement: Supplementary file 2 [file Image2.jpeg]

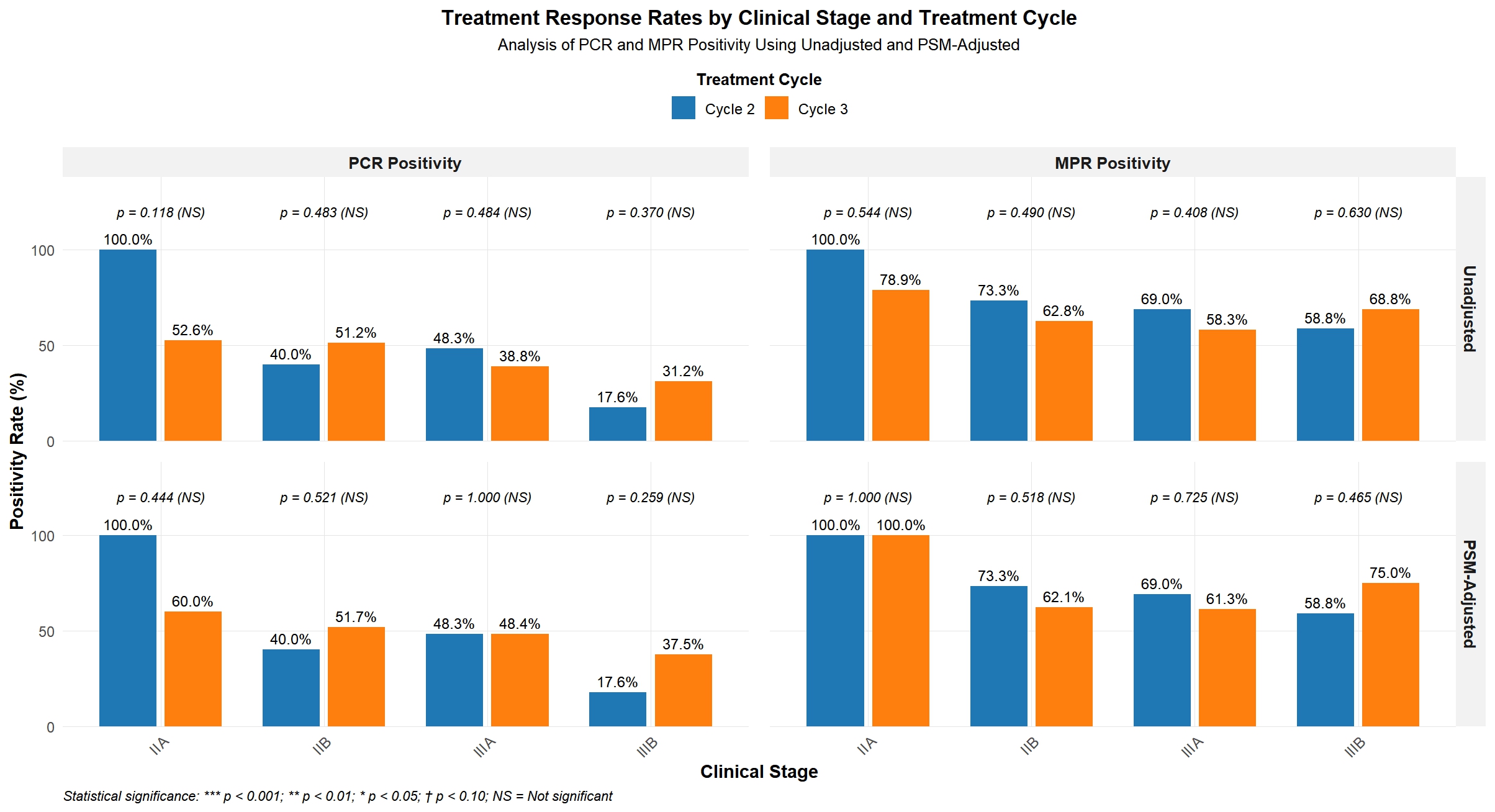

Supplement: Supplementary file 3 [file Image3.jpeg]

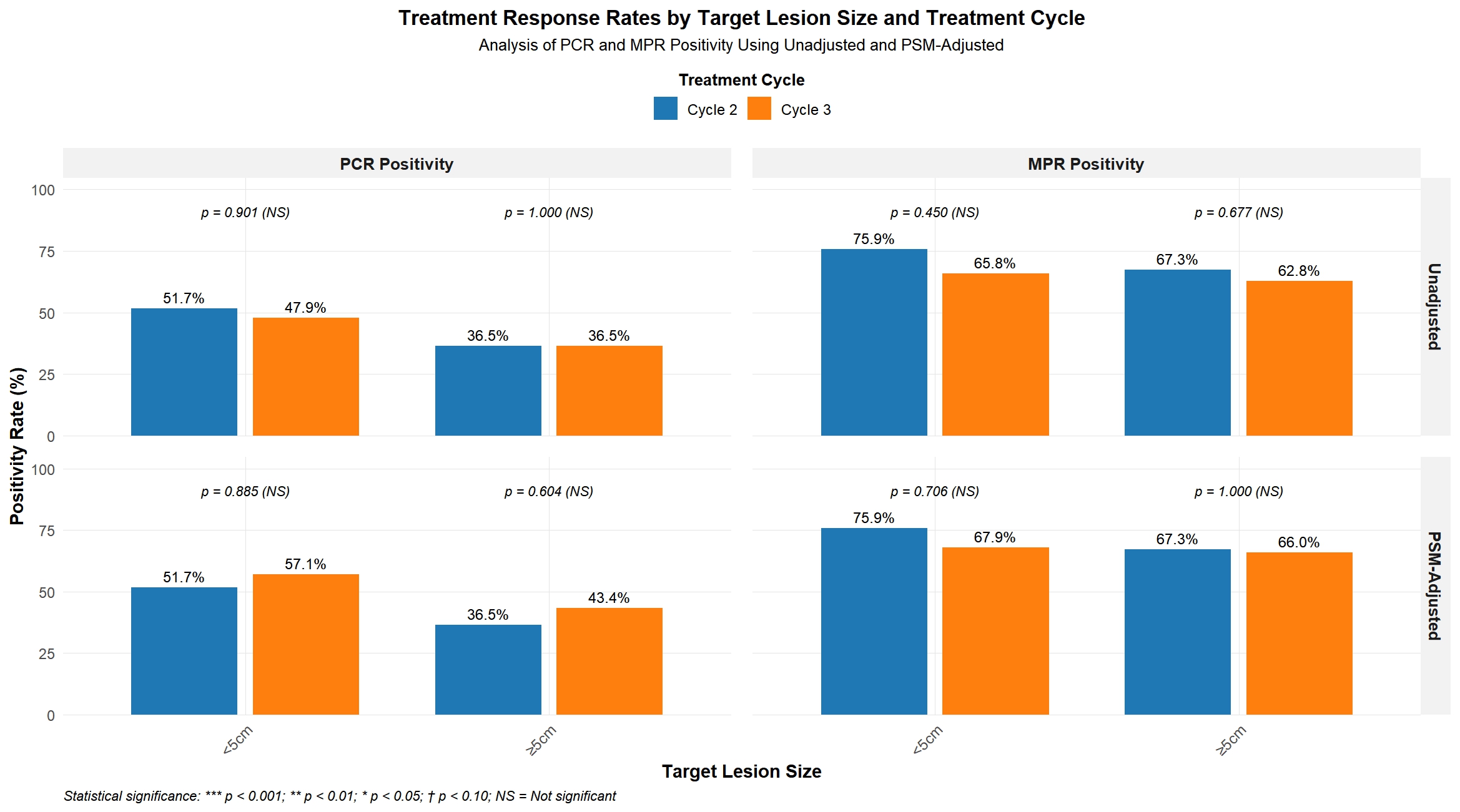

Supplement: Supplementary file 4 [file Image4.jpeg]
