# Supplementary material for: Two vs three cycles of neoadjuvant immunochemotherapy for resectable non-small-cell lung cancer: a real-world population-based study
Source: Front Immunol. 2025 Nov 17;16:1654830. doi: 10.3389/fimmu.2025.1654830 (PMC12665730; doi:10.3389/fimmu.2025.1654830)
Supplement: Supplementary file 5 [file Table1.docx]

Table S1：Comparison of Treatment-Related Adverse Events before and after propensity score matching adjustment.

|  | 2-cycle | 3-cycle | *P* | 2-cycle | 3-cycle | *P* |
| --- | --- | --- | --- | --- | --- | --- |
|  | Baseline | Baseline |  | PSM | PSM |  |
| Event | Any Grade | Any Grade |  | Any Grade | Any Grade |  |
| Treatment-Related Adverse Events |  |  |  |  |  |  |
| All | 40(49.4) | 119(52.0) | 0.881 | 40(49.4) | 40(49.4) | 1.000 |
| Anemia | 30(37) | 83(36.2) | 0.775 | 30(37.0) | 31(38.3) | 0.635 |
| Weight loss | 12(14.8) | 41(17.9) | 0.825 | 12(14.8) | 13(16.0) | 1.000 |
| Decreased neutrophils count | 5(6.2) | 38(16.6) | 0.032 | 5(6.2) | 14(17.3) | 0.056 |
| Increased Alanine aminotransferase/Alkaline phosphatase | 1(1.2) | 10(4.3) | 0.586 | 1(1.2) | 4(4.9) | 0.430 |
| Constipation | 0(0) | 1(0) | 1.000 | 0(0) | 1(1.2) | 1.000 |

Definition of Treatment-Related Adverse Events: Adverse events occurring between initiation of the first neoadjuvant therapy and immediately prior to surgical intervention (graded per CTCAE v4.0 criteria and coded using MedDRA v23.0 system).

Table S2：Comparison of Immune-Mediated Adverse Events before and after propensity score matching adjustment.

|  | 2-cycle | 3-cycle | *P* | 2-cycle | 3-cycle | *P* |
| --- | --- | --- | --- | --- | --- | --- |
|  | Baseline | Baseline |  | PSM | PSM |  |
| Event | Any Grade | Any Grade |  | Any Grade | Any Grade |  |
| Immune-Mediated Adverse Events |  |  |  |  |  |  |
| All | 15(18.5) | 24(10.5) | 0.093 | 15(18.5) | 8(9.9) | 0.177 |
| Pneumonitis | 12(14.8) | 21(9.2) | 0.228 | 12(14.8) | 7(8.6) | 0.329 |
| Hypothyroidism | 0(0) | 3(1.3) | 1.000 | 0(0) | 1(1.2) | 1.000 |
| Hyperthyroidism | 1(1.2) | 3(1.3) | 1.000 | 1(1.2) | 1(1.2) | 1.000 |
| Diabetes mellitus | 2(2.5) | 0(0) | 0.068 | 2(2.5) | 0(0) | 0.497 |

Definition of immune-related adverse events (irAEs): Events occurring between initiation of the first neoadjuvant therapy and immediately prior to surgical intervention meeting all criteria: 1) Investigator-assessed as potentially immune-mediated (irrespective of causality) .2) Graded per CTCAE v4.0 and coded per MedDRA v23.0 3) Absence of confirmed alternative etiology OR evidence of immune-mediated pathology.3) Due to the inherent limitations of this retrospective study, data pertaining to immune-related adverse events (irAEs) such as rash and nausea were not collected.
